# The efficacy of fat-free mass index and appendicular skeletal muscle mass index in cancer malnutrition: a propensity score match analysis

**DOI:** 10.3389/fnut.2023.1172610

**Published:** 2023-07-10

**Authors:** Wei Ji, XiangLiang Liu, Pengfei Liu, YuWei He, YiXin Zhao, Kaiwen Zheng, JiuWei Cui, Wei Li

**Affiliations:** ^1^Center of Cancer, The First Affiliated Hospital of Jilin University, Changchun, China; ^2^Cancer Department, Longyan First Hospital, Fujian, Longyan, China

**Keywords:** cancer, nutrition, malnutrition, skeletal muscle, prognosis

## Abstract

**Background:**

Reduced muscle mass (RMM) is a phenotypic criterion for malnutrition; the appendicular skeletal muscle mass index (ASMI) and fat-free mass index (FFMI) are both applicable indicators in the global leadership initiative on malnutrition (GLIM) guideline. However, their sensitivity and prognostic effect remain unclear.

**Methods:**

Clinical data of 2,477 patients with malignant tumors were collected. Multi-frequency bioelectrical impedance analysis was used to obtain ASMI and FFMI. RMM was confirmed by ASMI (< 7.0 kg/m^2^ for men and < 5.7 kg/m^2^ for women) or FFMI (< 17 kg/m^2^ for men and < 15 kg/m^2^ for women). Propensity score match analysis and logistic regression analysis were used to evaluate the efficacy of FFMI and ASMI in diagnosing severe malnutrition and multivariate Cox regression analysis to determine the efficacy of RMM in predicting survival.

**Results:**

In total, 546 (22.0%) and 659 (26.6%) participants were diagnosed with RMM by ASMI (RMM.ASMI group) and FFMI (RMM.FFMI group); 375 cases overlapped. Body mass index (BMI), midarm circumference, triceps skinfold thickness, and maximum calf circumference were all significantly larger in the RMM.FFMI group for both sexes (*P* < 0.05). A 1:1 matched dataset constructed by propensity score match contained 810 cases. RMM.FFMI was an influential factor of severe malnutrition with HR = 3.033 (95% CI 2.068–4.449, *P* < 0.001), and RMM.ASMI was a predictive factor of overall survival (HR = 1.318, 95% CI 1.060–1.639, *P* = 0.013 in the RMM.ASMI subgroup, HR = 1.315, 95% CI 1.077–1.607, *P* = 0.007 in the RMM.FFMI subgroup).

**Conclusion:**

In general, RMM indicates negative clinical outcomes; when defined by FFMI, it predicts nutritional status, and when defined by ASMI, it is related to poor survival in cancer patients.

## 1. Introduction

In 2018, the number of cancer patients and cancer-related deaths increased by 18.1 million and 9.6 million globally, respectively (1). According to previous research, the incidence of malnutrition in patients with malignant cancers is 15–40% at initial diagnosis and up to 40–80% during treatment (2). Malnutrition leads to functional decline, reduces the quality of life, increases hospital costs, and even causes mortality, which explains why it has become the focus of recent research interest (3).

Cancer-related malnutrition results in changes in body composition, mainly muscle depletion, and deteriorating biological function. A decreasing food intake or absorption and inflammation are the main reasons (4–7). In fact, 55% of patients reported reduced dietary intake after suffering from cancer (8). Inflammation, a hallmark of cancer, is involved in malnutrition through multiple mechanisms (9, 10). Interleukin-1 (IL-1), IL-6, tumor necrosis factor-alpha (TNF-α), and interferon-γ are demonstrated to contribute to anorexia (11, 12). For instance, Han et al. (13) reported that IL-6 and TNF-α could regulate white adipose tissue lipolysis browning, resulting in the development of malnutrition. Negative nitrogen balance and muscle wasting are significant characteristics of cancer-related malnutrition (14). In addition, IL-6 overexpression increases muscle proteolysis through both ubiquitin-dependent and autophagy-related pathways (15) and can affect mitochondrial dynamics, increasing the oxidative metabolism of skeletal muscle (16).

Cancer-related malnutrition is related to frequent use of antibiotics and long hospitalization, resulting in decreased quality of life and increased cost and psychological pressure (17). Accordingly, screening and assessment of malnutrition are important. The patient-generated subjective global assessment (PG-SGA) is the gold standard in evaluating the nutritional status of cancer patients (18). However, in 2018, the Global Clinical Nutrition Community released a consensus proposing a global screening and diagnostic guideline on malnutrition called the global leadership initiative on malnutrition (GLIM), which includes phenotypic and etiologic criteria (19). Muscle reduction is one of the phenotypic criteria. Both the appendicular skeletal muscle mass index (ASMI) and fat-free mass index (FFMI) are parameters used to evaluate muscle mass, and their cutoff values depend on ethnicities and evaluation tools. However, the diagnostic sensitivity and prognostic effectiveness of the two parameters have not been fully compared. Hence, the study was designed to clarify this point. In this study, we included 2,477 cancer cases. Taking PG-SGA as a gold standard, the diagnostic values of ASMI and FFMI were compared by propensity score match analysis. In addition, the prognostic effectiveness of the two parameters was compared considering overall survival (OS) as an endpoint.

## 2. Patients and methods

The study protocol adhered to the Declaration of Helsinki and was approved by the Ethics Committee of the First Hospital of Jilin University (2017-362).

### 2.1. Patients

The clinical data of patients with malignant tumors admitted to the First Affiliated Hospital of Jilin University from November 2011 to December 2018 were collected. Inclusion criteria were as follows: (1) age > 18 years old and (2) pathological diagnosis of malignant tumors. Exclusion criteria were as follows: (1) ≥2 coexisting types of tumors; (2) suffering from server pleural effusion and/or ascites; (3) under regular hemodialysis; or (4) death within 3 days after admission.

Clinical data for each participant were collected by trained personnel. Laboratory examinations, anthropometric measurements, and bioelectric impedance analysis (BIA) were completed within 3 days of admission. Operating details are displayed in Supplementary material 1. Data included the following: (1) General characteristics: age, sex, smoking history, alcohol drinking, comorbidities (diabetes and hypertension), tumor site (the lung, digestive tract, liver, breast, and gynecological), and metastasis. (2) Laboratory examinations: serum albumin concentration, serum C-reaction protein (CRP), leukocyte, neutrophils, lymphocytes, platelets, neutrophils to lymphocytes ratio (NLR), platelets to lymphocytes ratio (PLR), and systematic inflammation index (SII). (3) Evaluation scales: PG-SGA. (4) Anthropometric measurements: body mass index (BMI), mid-arm circumference (MAC), triceps skinfold thickness (TSF), maximum calf circumference (CC), and hand-grip strength (HGS). (5) BIA indices: measured by a multi-frequency bioelectrical impedance body composition analyzer (InbodyS10; Biospace Co.^®^). Both ASM and FFM were recorded. (6) Survival data: OS was recorded from diagnosis to mortality due to any cause. The corresponding formulas used are as follows:


$$\begin{array}{l} BMI=weight\left(kg\right)/height^{2}\;\left(m^{2}\right)\\ FFMI=FFM/height\;\left(m^{2}\right)\\ ASMI\left(kg/m^{2}\right)=ASM/height^{2}\;\left(m^{2}\right)\\ SII=\left(platelets\times neutrophils/lymphocytes\right)/1,000 \end{array}$$

### 2.2. Reduced muscle mass

GLIM recommends measurement by dual-energy absorptiometry or other validated body composition measures including BIA for detecting reduced muscle mass (RMM). In this study, RMM was confirmed based on ASMI (< 7.0 kg/m^2^ for men and < 5.7 kg/m^2^ for women) or FFMI (< 17 kg/m^2^ for men and < 15 kg/m^2^ for women) as measured by BIA.

### 2.3. Statistical analysis

Data were analyzed using SPSS for Windows version 26.0 (IBM SPSS Statistics, IBM Corp., Armonk, NY) and R version 4.0 (R Foundation for Statistical Computing, Vienna, Austria).

A Venn plot was drawn to depict the overlap and division of RMM diagnosed by FFMI and ASMI. The Kolmogorov–Smirnov test was used to confirm normal distributions of continuous data. An independent *t*-test was used for normally distributed data. Counting data were analyzed using the chi-square test, and the *z*-test with Bonferroni adjustment was adopted for multiple comparisons. Next, propensity score match (PSM) analysis was performed. Multicollinearity was tested by linear regression analysis; a variance inflation factor (VIF) >10 was considered to indicate collinearity. Conditional logistic regression analysis was adopted to evaluate the efficacy of FFMI and ASMI in diagnosing severe malnutrition (PG-SGA ≥ 9). The shared frailty model for survival analysis was then performed to determine the efficacy of RMM in predicting survival benefit. A *P*-value of < 0.05 was considered to indicate statistical significance.

## 3. Results

### 3.1. RMM detected by ASMI and FFMI

**Figure 1:**
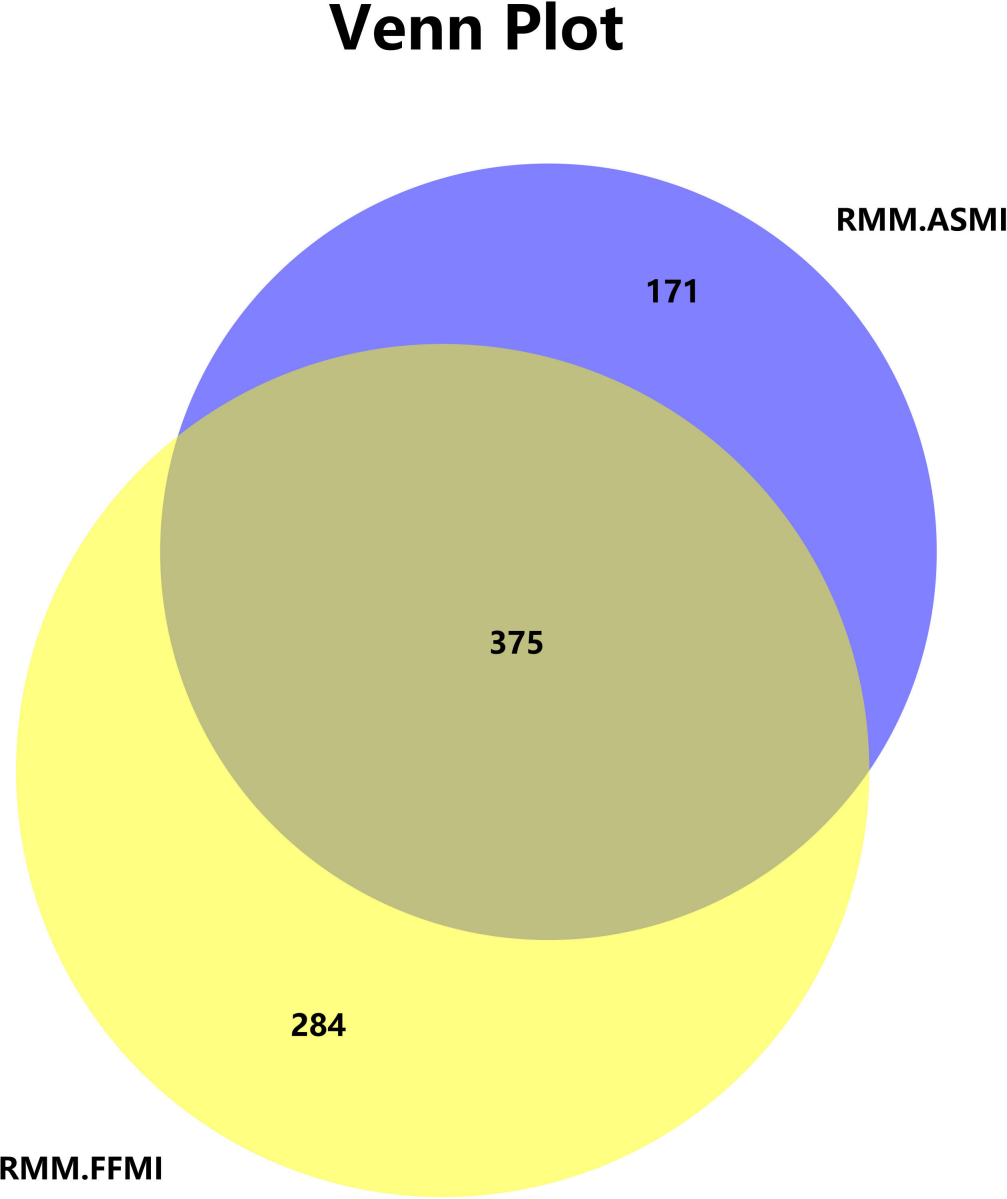
RMM diagnosed with ASMI and FFMI. RMM, reduced muscle mass; ASMI, appendicular skeletal muscle mass index; FFMI, fat-free mass index.

Among the 2,477 participants involved, 546 (22.0%) and 659 (26.6%) participants were diagnosed with RMM by ASMI (RMM.ASMI group) or FFMI (RMM.FFMI group), respectively. There was an overlap of 375 cases, comprising 68.7% of the RMM.ASMI group and 56.9% of the RMM.FFMI group (Figure 1). In total, 33.3% of patients in the RMM.ASMI group were men, significantly less than in the RMM.FFMI group (46.9%, *P* < 0.001). Age, smoking history, drinking history, comorbidities, tumor sites, and metastasis did not differ between the groups (*P* > 0.05) (Supplementary material 2).

### 3.2. Characteristics of RMM as defined by AMSI and FFMI

No significant difference was detected in albumin, CRP, leukocytes, neutrophils, lymphocytes, platelets, NLR, PLR, and SII between the groups (*P* > 0.05). Then, the anthropometric measurements were compared after stratifying by sex. BMI, MAC, TSF, and CC were all significantly larger in the RMM.FFMI group for both sexes (*P* < 0.05) (Table 1). HGS did not differ between the groups in both sexes (*P* > 0.05).

**Table 1:** Serum nutrition, inflammation indices, and anthropometric measurements of RMM defined by ASMI and FFMI (mean ± SD)/[*n* (%)].

**Variables**	**RMM**		***t***	***P***
	**ASMI**	**FFMI**		
Albumin (g/L)	37.84 ± 5.27	38.24 ± 5.26	−1.325	0.186
CRP (mg/L)	19.93 ± 35.75	19.25 ± 32.62	0.276	0.782
Leukocyte (^*^10^9^/L)	6.94 ± 3.97	6.76 ± 3.08	0.829	0.407
Neutrophils (^*^10^9^/L)	4.89 ± 3.81	4.70 ± 3.22	0.953	0.341
Lymphocytes (^*^10^9^/L)	1.53 ± 0.69	1.96 ± 0.80	−1.017	0.309
Platelets (^*^10^9^/L)	243.03 ± 97.28	240.82 ± 93.35	0.401	0.689
NLR	4.11 ± 4.40	3.89 ± 4.33	0.886	0.376
PLR	188.51 ± 110.87	184.25 ± 151.30	0.546	0.585
SII^#^	1,020.45 ± 466.66	939.08 ± 181.74	1.064	0.288
**Female**				
BMI (kg/m^2^)	19.60 ± 1.89	20.53 ± 2.76	−5.210	< 0.001
MAC (cm)	23.92 ± 2.59	24.62 ± 3.10	−3.282	0.001
TSF (mm)	15.14 ± 5.52	16.79 ± 6.21	−3.740	< 0.001
CC (cm)	30.54 ± 3.14	31.19 ± 3.38	−2.684	0.007
HGS (kg)	17.52 ± 6.12	17.50 ± 6.01	0.033	0.974
**Male**				
BMI (kg/m^2^)	18.63 ± 1.14	20.11 ± 2.07	−10.222	< 0.001
MAC (cm)	23.64 ± 2.14	24.66 ± 2.58	−4.718	< 0.001
TSF (mm)	11.33 ± 4.54	13.44 ± 5.31	−4.662	< 0.001
CC (cm)	30.82 ± 3.14	32.07 ± 3.87	−3.653	< 0.001
HGS (kg)	26.03 ± 7.75	26.89 ± 7.73	−1.199	0.231

^#^SII = platelets^*^neutrophils/lymphocytes. RMM, reduced muscle mass; ASMI, appendicular skeletal muscle mass index; FFMI, fat-free mass index; CRP, C-reaction protein; NLR, neutrophils to lymphocytes ratio; PLR, platelets to lymphocytes ratio; SII, systematic inflammation index; MAC, mid-arm circumference; TSF, triceps skinfold thickness; CC, maximum calf circumference; HGS, hand-grip strength.

### 3.3. Malnutrition in the RMM.ASMI and RMM.FFMI groups

A 1:1 matched dataset was constructed by PSM for further analysis. Baseline information (age, smoking history, drinking history, comorbidities, tumor sites, and metastasis) was matched considering severe malnutrition (PG-SGA ≥ 9) as a dependent variable. The matched dataset contained 810 cases; all basic characteristics were comparable (*P* > 0.05) (Table 2; Supplementary material 3). Leukocyte data were excluded due to collinearity (Supplementary material 4). A conditional logistic regression showed that RMM.ASMI was not an influential factor in the univariate model (*P* = 0.122). However, RMM.FFMI was an influential factor of severe malnutrition even in the multivariate model, with HR = 4.070 (95% CI 2.753–6.019, *P* < 0.001) after adjusting all involved characteristics (Table 3).

**Table 2:** Characteristics of the involved population before and after propensity score matching.

**Variables**	**Before**			**After**		
	**PG-SGA 0–8**	**PG-SGA** ≥**9**	***P***	**PG-SGA 0–8** ***n** =* **405**	**PG-SGA** ≥ **9** ***n** =* **405**	***P***
Age (year)			< 0.001			0.880
< 65	1,661 (81.0)	286 (67.1)		276 (68.1)	274 (67.7)	
≥65	390 (19.0)	140 (32.9)		129 (31.9)	131 (32.3)	
Sex			< 0.001			0.779
Male	805 (39.1)	214 (50.2)		206 (50.9)	202 (49.9)	
Female	1,246 (60.8)	212 (49.8)		199 (49.1)	203 (50.1)	
Smoking			0.001			0.888
Yes	782 (38.1)	200 (46.9)		221 (54.6)	219 (54.1)	
No	1,269 (61.9)	226 (53.1)		184 (45.4)	186 (45.9)	
Drinking			0.050			0.933
Yes	369 (18.0)	94 (22.1)		91 (22.5)	90 (22.2)	
No	1,682 (82.0)	332 (77.9)		314 (77.5)	315 (77.8)	
Comorbidity			0.887			0.270
No	1,939 (79.9)	336 (78.9)		316 (78.0)	321 (79.3)	
Hypertension	308 (15.0)	67 (15.7)		57 (14.1)	63 (15.6)	
Diabetes	104 (5.1)	23 (5.4)		32 (7.9)	21 (5.2)	
Tumor site			< 0.001			0.464
Lung	740 (36.1)a	128 (30.0)b		126 (31.1)	120 (29.6)	
Digestive tract	398 (19.4)a	178 (41.8)b		180 (44.4)	172 (42.5)	
Liver	111 (5.4)a	55 (12.9)b		35 (8.6)	51 (12.6)	
Breast	660 (32.2)a	39 (9.2)b		41 (10.1)	37 (9.1)	
Gynecology	142 (6.9)a	26 (6.1)a		23 (5.7)	25 (6.2)	
Metastasis			< 0.001			0.533
M0	1,529 (78.7)	286 (70.6)		294 (72.6)	286 (70.6)	
M1	415 (21.3)	119 (29.4)		111 (27.4)	119 (29.4)	

PG-SGA, Patient-Generated Subjective Global Assessment.

**Table 3:** Conditional logistic regression analysis for malnutrition.

**Variables**	**Univariable**		**Multivariable**	
	**OR (95%CI)**	***P***	**OR (95%CI)**	***P***
Albumin (g/L)	0.923 (0.868–0.982)	< 0.001	0.937 (0.906–0.968	< 0.001
CRP (mg/L)	1.004 (0.994–1.014)	0.452		
Neutrophils (^*^10^9^/L)	1.391 (1.024–1.889)	0.035		
Lymphocytes (^*^10^9^/L)	0.763 (0.418–1.393)	0.379		
Platelets (^*^10^10^/L)	1.002 (0.995–1.009)	0.595		
NLR	0.877 (0.656–1.174)	0.378		
PLR	1.007 (0.998–1.016)	0.109		
SII.Model^#^	0.905 (0.819–1.000)	0.049		
BMI (kg/m^2^)	1.003 (0.850–1.183)	0974		
MAC (cm)	0.963 (0.840–1.103)	0.586		
TSF (mm)	1.024 (0.985–1.066)	0.231		
CC (cm)	0.954 (0.855–1.065)	0.404		
HGS (kg)	0.972 (0.935–1.011)	0.158		
RMM.ASMI	2.094 (0.821–5.343)	0.122		
RMM.FFMI	3.675 (1.627–8.302)	0.002	4.070 (2.753–6.019)	< 0.001

^#^SII.Model=SII/100, which is calculated merely for model input given practical clinical practice. CRP, C-reaction protein; NLR, neutrophils to lymphocytes ratio; PLR, platelets to lymphocytes ratio; SII, systematic inflammation index; BMI, body mass index; MAC, mid-arm circumference; TSF, triceps skinfold thickness; CC, maximum calf circumference; HGS, hand-grip strength; RMM, reduced muscle mass; ASMI, appendicular skeletal muscle mass index; FFMI, fat-free mass index. *means multiplication.

### 3.4. Efficacy of RMM in survival prediction

To determine the efficacy of RMM as detected by ASMI and FFMI, survival analysis was performed in the whole 2,477 cases, followed by sensitivity analysis in subgroups. In the general population, RMM.ASMI was a predictive factor (HR = 1.301, 95% CI 1.034–1.635, *P* = 0.025) in the univariate Cox regression analysis but not in the multivariate analysis (Forward: Wald). Apart from the baseline factors, albumin (HR = 0.953, 95% CI 0.924–0.982, *P* = 0.001), neutrophils (HR = 1.071, 95% CI 1.020–1.126, *P* = 0.006), and TSF (HR = 0.959, 95% CI 0.936–0.982, *P* = 0.001) were considered as influential factors of OS (Table 4).

**Table 4:** Cox regression analysis for overall survival in the general population.

**Variables**	**Univariable**		**Multivariable**	
	**OR (95%CI)**	***P***	**OR (95%CI)**	***P***
Age (year)	1.176 (0.926–1.494)	0.184		
Sex	0.763 (0.606–0.962)	0.022		
Smoking	1.453 (1.155–1.827)	0.001		
Drinking	1.050 (0.801–1.377)	0.724		
**Comorbidity**				
Hypertension	1.092 (0.798–1.496)	0.582		
Diabetes	1.156 (0.732–1.826)	0.535		
**Tumor site**				
Digestive tract	0.421 (0.324–0.547)	< 0.001	0.524 (0.367–0.748)	< 0.001
Liver	1.050 (0.750–1.470)	0.777	1.149 (0.728–1.813)	0.552
Breast	0.156 (0.079–0.306)	< 0.001	0.450 (0.193–1.051)	0.065
Gynecology	0.233 (0.114–0.476)	< 0.001	0.414 (0.166–1.031)	0.058
Metastasis	3.745 (2.967–4.728)	< 0.001	3.349 (2.443–4.592)	< 0.001
Albumin (g/L)	0.951 (0.931–0.973)	< 0.001	0.953 (0.924–0.982)	0.001
CRP (mg/L)	1.005 (1.002–1.009)	0.003		
Neutrophils (^*^10^9^/L)	1.058 (1.023–1.094)	0.001	1.071 (1.020–1.126)	0.006
Lymphocytes (^*^10^9^/L)	0.833 (0.704–0.986)	0.033		
Platelets (^*^10^10^/L)	1.016 (1.004–1.027)	0.008		
NLR	1.001 (0.993–1.009)	0.815		
PLR	1.000 (1.000–1.001)	0.058		
SII.Model^#^	1.002 (0.999–1.006)	0.226		
BMI (kg/m^2^)	0.928 (0.894–0.962)	< 0.001		
MAC (cm)	0.939 (0.905–0.973)	0.001		
TSF (mm)	0.960 (0.941–0.979)	< 0.001	0.959 (0.936–0.982)	0.001
CC (cm)	0.962 (0.933–0.992)	0.012		
HGS (kg)	0.995 (0.983–1.007)	0.420		
RMM.ASMI	1.301 (1.034–1.635)	0.025		
RMM.FFMI	1.141 (0.905–1.440)	0.265		

^#^SII.Model=SII/100, which is calculated merely for model input given practical clinical practice. CRP, C-reaction protein; NLR, neutrophils to lymphocytes ratio; PLR, platelets to lymphocytes ratio; SII, systematic inflammation index; BMI, body mass index; MAC, mid-arm circumference; TSF, triceps skinfold thickness; CC, maximum calf circumference; HGS, hand-grip strength; RMM, reduced muscle mass; ASMI, appendicular skeletal muscle mass index; FFMI, fat-free mass index.

**Figure 2:**
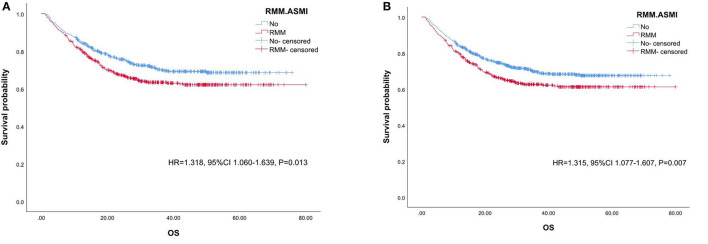
Kaplan–Meier survival analysis of RMM.ASMI in OS. **(A)**. the RMM.ASMI subgroup, **(B)**. the RMM.FFMI subgroup. RMM, reduced muscle mass; ASMI, appendicular skeletal muscle mass index; FFMI, fat-free mass index; OS, overall survival.

Next, two 1:1 matched subgroups were constructed, which were adjusted by all baseline factors (age, smoking history, drinking history, comorbidities, tumor sites, and metastasis) and took RMM.FFMI and RMM.ASMI as the dependent variables, respectively. The RMM.ASMI subgroup contained 1,034 cases and the RMM.FFMI subgroup contained 1,232 cases. Since the baseline characteristics were already matched, shared frailty survival analysis was performed in each subgroup. As shown in Figure 2, RMM.ASMI behaved as a predictive factor of OS in both subgroups (HR = 1.318, 95% CI 1.060–1.639, *P* = 0.013 in the RMM.ASMI subgroup, HR = 1.315, 95% CI 1.077–1.607, *P* = 0.007 in the RMM.FFMI subgroup) but RMM.FFMI did not in any subgroup (*P* > 0.05). Thus, Cox regression was performed in the subgroups to further clarify the role of RMM.ASMI in predicting OS (Table 5). In both subgroups, only albumin (HR = 0.961, 95% CI 0.931–0.992, *P* = 0.015; HR = 0.941, 95% CI 0.912–0.970, *P* < 0.001) and platelets (HR = 1.004, 95% CI 1.002–1.006, *P* = 0.026; HR = 1.003, 95% CI 1.002–1.004, *P* = 0.002) were maintained in the Cox regression model. RMM.ASMI was not retained in both models.

**Table 5:** Cox regression analysis for overall survival in subgroups^#^.

**Variables**	**RMM.ASMI**		**RMM.FFMI**	
	**OR (95%CI)**	***P***	**OR (95%CI)**	***P***
Albumin (g/L)	0.961 (0.931–0.992)	0.015	0.941 (0.912–0.970)	< 0.001
Platelets (^*^10^10^/L)	1.004 (1.002–1.006)	0.026	1.003 (1.002–1.004)	0.002

^#^CRP, Neutrophils, Lymphocytes, NLR, PLR, SII, BMI, MAC, TSF, CC, HGS, RMM.ASMI, and RMM.FFMI were also involved as covariates but not displayed in the table since they were not retained in the multivariable Cox regression models (*P* > 0.05). CRP, C-reaction protein; NLR, neutrophils to lymphocytes ratio; PLR, platelets to lymphocytes ratio; SII, systematic inflammation index; BMI, body mass index; MAC, mid-arm circumference; TSF, triceps skinfold thickness; CC, maximum calf circumference; HGS, hand-grip strength; RMM, reduced muscle mass; ASMI, appendicular skeletal muscle mass index; FFMI, fat-free mass index.

## 4. Discussion

RMM, measured by FFMI and ASMI, was considered a phenotypic criterion for malnutrition in the GLIM guideline. The prevalence of RMM diagnosed by FFMI and ASMI was 22.0 and 26.2%, respectively, with approximately three-fifths overlapping. As shown by sensitive analysis, FFMI was significant in diagnosing severe malnutrition. However, in survival analysis, although the effect of ASMI was always significant in univariate regression, neither FFMI nor ASMI was retained in multivariate regressions. These results are due to intrinsic differences between FFMI and ASMI and implied their discrimination in clinical utility, which should be paid attention.

Chronic inflammation and depletion accompany cancer over the whole process (20). Infection and non-infectious inflammation are the initial stages of malignant lesions (21), with persistent crosstalk between inflammation and cancer, mainly converging at the level of the transcription factors such as signal transducer and activator of transcription 3 (STAT3) and nuclear factor-κB (NF-κB). Downstream cytokines including IL-6, TNF-α, and TGF-beta also deteriorate energy and protein metabolism (22). The increased metabolism and deteriorated catabolism induce changes in body composition, especially to the muscle tissue, and induce even the occurrence of sarcopenia and cachexia. In addition, treatment-related adverse events, especially nausea, vomiting, and other gastrointestinal symptoms, aggravate the situation.

The FFMI is calculated based on the FFM, which represents the body composition except fat including muscle and bone mass, and organs such as the liver. The ASMI, calculated based on the ASM, merely refers to the skeletal muscle mass in the limbs. Thus, the FFM is more consistent with the weight and BMI, as supported by the present results. PG-SGA is the gold standard of malnutrition assessment for cancer patients, based on weight loss, symptoms, activities and function, metabolic demand (largely refers to inflammation), and physical examinations. As a part of the latter, the muscle assessment includes parts of the torso such as the temples (temporalis muscle), clavicle (pectoralis and deltoids), shoulders (deltoids), interosseous muscles, scapula (latissimus dorsi, trapezius, and deltoids), and a small part of the muscles in the thigh (quadriceps) and calf (gastrocnemius). Therefore, RMM as measured by FFMI closely relates to the severe malnutrition detected by PG-SGA ≥ 9. ASM can be persevered by physical activity, especially resistance exercises, as recommended by the guidelines in sarcopenia (23). A detectable ASM loss implies more severe exhaustion. In the updated guidelines, HGS, the functional parameter of ASM, has a higher priority than absolute ASM in diagnosing sarcopenia. HGS was significantly, and similarly, reduced in both RMM groups diagnosed by FFMI and ASMI. However, BMI, MAC, TSF, and CC were all significantly lower in the RMM.ASMI group for both sexes, which suggested that ASM loss indicates a worse situation of depletion. That is why the RMM diagnosed by ASMI and not the RMM diagnosed by FFMI is an influential factor in survival.

However, the decreased ASMI was not included in the multivariate regression model. Parameters such as platelets, neutrophils, TSF, and BMI were unstable in a sensitivity analysis. In contrast, albumin always contributed to nutritional status and survival. Gupta et al. (24) performed a systematic review and found that albumin was of predictive value in survival in various cancer types. Albumin, accounting for approximately 50% of the total protein content, is the most common clinical indicator of nutritional status and is involved in the inflammatory response, acting as an acute-phase protein (25, 26). In addition, serum albumin allows a simple estimation of visceral protein function. Suppressed albumin synthesis is partly due to the activation of cytokines such as IL-1, IL-6, and TNF-α (27), a common observation in cancer, resulting in hypoalbuminemia. This increases the demand for certain amino acids, which, in case of inadequate dietary intake, may mobilize the breakdown of skeletal muscle (28). Alternatively, the oxidative stress induced by cytokines may increase the permeability of the microvascular barriers, thus allowing an increased albumin leakage through capillaries (29, 30). Furthermore, the presence of metastatic tumor cells in the liver may induce the Kupffer cells to produce inflammatory cytokines and chemokines, which foster monocyte infiltration into the liver. These may modulate albumin synthesis by hepatocytes and support tumor development by angiogenesis and T-cell suppression (31). Thus, albumin levels can serve as good indicators of nutritional status and cancer prognosis.

There are some limitations to this study. First, selection bias might exist because this was a retrospective study and the demand for complete clinical data. Second, despite adopting PSM and sensitive analyses, the value of unstable parameters such as platelets, neutrophils, TSF, and BMI requires further analysis to provide a confidential reference.

In conclusion, this study revealed that RMM indicates negative clinical outcomes and highlighted the intrinsic differences between FFM and ASM, suggesting the need for rational choice in clinical practices to support future decision-making in cancer patients. More importantly, RMM as defined by FFMI predicts nutritional status, whereas when defined by ASMI, it is related to poor survival in cancer patients. In addition, serum albumin appeared to be an influential factor for both malnutrition and survival. The instability of other parameters revealed by sensitive analysis reminds clinical practitioners of precious opinions on these parameters.

## Data availability statement

The raw data supporting the conclusions of this article will be made available by the authors, without undue reservation.

## Ethics statement

The studies involving human participants were reviewed and approved by the Ethics Committee of the First Hospital of Jilin University. The patients/participants provided their written informed consent to participate in this study.

## Author contributions

All authors made a significant contribution to the study reported, whether that is in the conception, study design, execution, acquisition of data, analysis, and interpretation, or all these areas took part in drafting, revising, or critically reviewing the article gave final approval of the version to be published have agreed on the journal to which the article has been submitted and agreed to be accountable for all aspects of the study.
