# Supplementary material for: The efficacy of fat-free mass index and appendicular skeletal muscle mass index in cancer malnutrition: a propensity score match analysis
Source: Front Nutr. 2023 Jul 10;10:1172610. doi: 10.3389/fnut.2023.1172610 (PMC10364448; doi:10.3389/fnut.2023.1172610)
Supplement: Supplementary file 1 [file Data_Sheet_1.docx]

Suppl.1 Operating details of anthropometric measurements and bioelectrical impedance analysis (BIA)

**Body mass index (BMI)** Patients needed to empty their urines and fast for 2 hours before the measurement. During the measurement, patients took off their shoes and wore light clothing. Height and weight were measured accurate to 1cm and 0.1kg. BMI (kg/m^2^) was calculated by weight (kg)/height (m) ^2^.

**Then mid‐arm circumference (MAC), skinfold thickness (TSF) and the mid-arm muscle circumference (MAMC)** MAC of the non-dominant side was measured to the nearest 0.5cm using a nonelastic tape when patients were standing. TSF at the same place was measured accurate to 1mm using vernier calipers. MAMC (cm) was calculated by MAC (cm)-π x [TSF (mm)/10].

**Calf circumference (CC)** The maximum circumference of the left calf was measured with a nonelastic tape when standing, which accurate to 0.5cm.

**Hand grip strength (HGS)** Patients seated with 90° elbow flexion. The grip strength of the dominant hand was measured to the nearest 1kg at least twice with the Jamar dynamometer, and the maximum reading was recorded.

**BIA** The multi-frequency bioelectrical impedance body composition analyzer InbodyS10 (BiospaceCo ®) was adopted. Patients needed to empty their urines, fast for 2 hours and keep quiet before the measurement. During the measurement, patients wore light clothing and contacted with 8 electrodes. Relevant parameters were measured or calculated according to the formula of InbodyS10.

Suppl.2 General characteristics of involved patients [n (% of column)]

| Variables | RMM | | *χ*^2^ | *P* | |  |
| --- | --- | --- | --- | --- | --- | --- |
|  | ASMI | FFMI |  | |  | |
| Age (year) |  |  | 0.644 | | 0.422 | |
| < 65 | 350(61.4) | 437(66.3) |  | |  | |
| ≥ 65 | 196(35.9) | 222(33.7) |  | |  | |
| Sex |  |  | 22.727 | | < 0.001 | |
| Male | 182(33.3) | 309(46.9) |  | |  | |
| Female | 364(66.7) | 350(53.1) |  | |  | |
| Smoking |  |  | 1.786 | | 0.181 | |
| Yes | 235(43.0) | 309(46.9) |  | |  | |
| No | 311(57.0) | 230(53.1) |  | |  | |
| Drinking |  |  | 1.717 | | 0.190 | |
| Yes | 87(15.9) | 124(18.8) |  | |  | |
| No | 459(84.1) | 535(81.2) |  | |  | |
| Comorbidity |  |  | 0.904 | | 0.636 | |
| No | 463(84.8) | 556(84.4) |  | |  | |
| Hypertension | 57(10.4) | 64(9.7) |  | |  | |
| Diabetes | 26(4.8) | 39(5.9) |  | |  | |
| Tumor site |  |  | 3.549 | | 0.470 | |
| Lung | 188(34.3) | 250(37.9) |  | |  | |
| Digestive tract | 190(34.8) | 214(32.5) |  | |  | |
| Liver | 43(7.9) | 56(8.5) |  | |  | |
| Breast | 89(16.3) | 108(16.4) |  | |  | |
| Gynecology | 36(6.6) | 31(4.7) |  | |  | |
| Metastasis |  |  | 0.196 | | 0.658 | |
| M0 | 374(72.3) | 439(71.2) |  | |  | |
| M1 | 143(27.7) | 178(28.8) |  | |  | |

RMM: reduced muscle mass; ASMI: appendicular skeletal muscle mass index; FFMI: fat-free mass index.

Suppl.3 The propensity score analysis


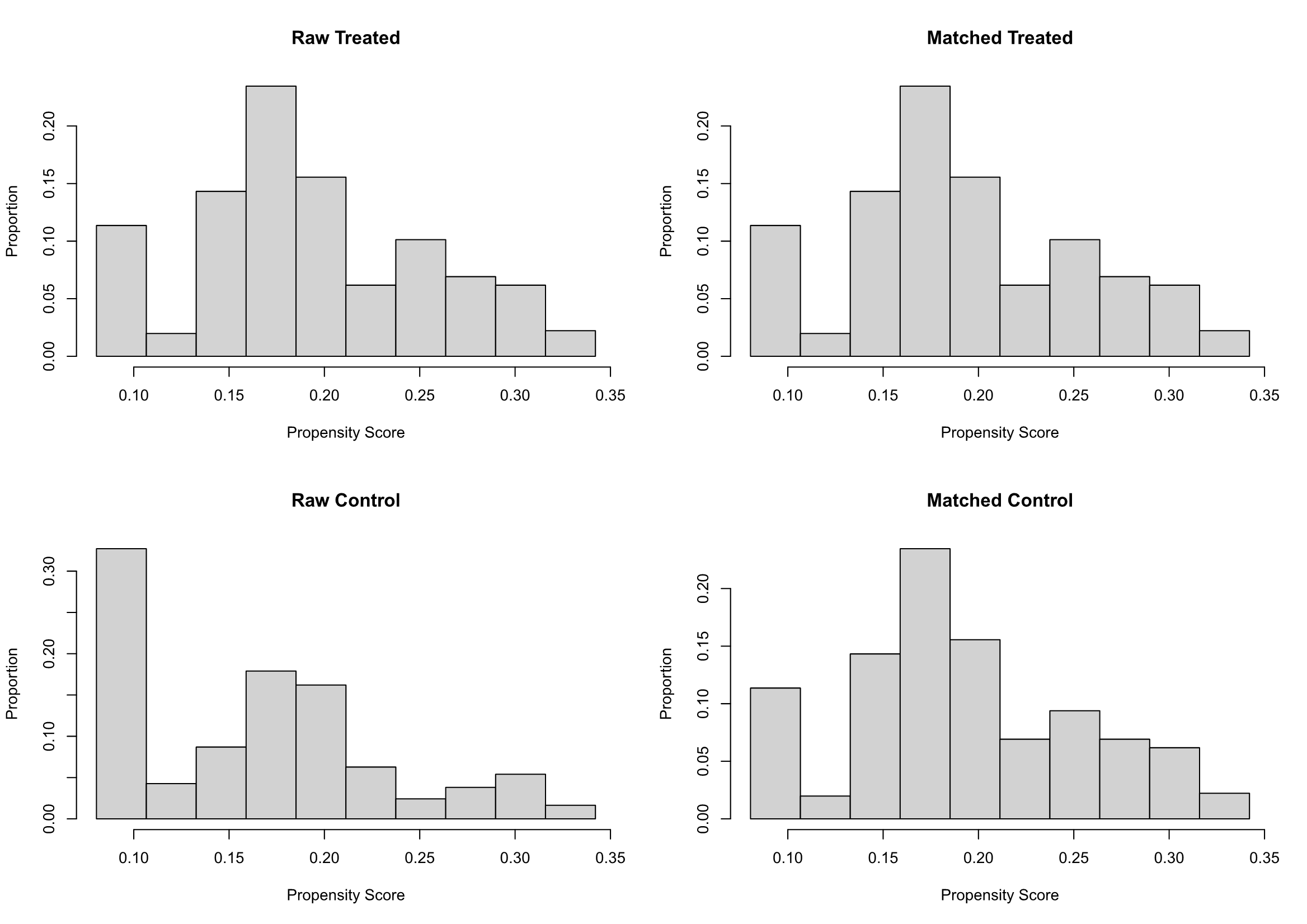


Suppl.4 Collinearity diagnosis

| Variables | Before | |  | After | |
| --- | --- | --- | --- | --- | --- |
|  | Tolerance | VIF |  | Tolerance | VIF |
| Age (year) | 0.800 | 1.250 |  | 0.801 | 1.249 |
| Sex | 0.406 | 2.463 |  | 0.406 | 2.463 |
| Smoking | 0.614 | 1.628 |  | 0.630 | 1.587 |
| Drinking | 0.642 | 1.557 |  | 0.645 | 1.551 |
| Comorbidity | 0.910 | 1.099 |  | 0.910 | 1.098 |
| Tumor site | 0.720 | 1.389 |  | 0.720 | 1.389 |
| Metastasis | 0.781 | 1.280 |  | 0.786 | 1.273 |
| Albumin (g/L) | 0.161 | 6.230 |  | 0.737 | 1.356 |
| CRP (mg/L) | 0.093 | 10.727 |  | 0.694 | 1.440 |
| Leukocyte (*10^9^/L) | 0.965 | 1.036 |  |  |  |
| Neutrophils (*10^9^/L) | 0.402 | 2.485 |  | 0.141 | 7.068 |
| Lymphocytes (*10^9^/L) | 0.737 | 1.357 |  | 0.966 | 1.035 |
| Platelets (*10^9^/L) | 0.673 | 1.486 |  | 0.416 | 2.405 |
| NLR | 0.101 | 9.870 |  | 0.101 | 9.866 |
| PLR | 0.175 | 5.700 |  | 0.181 | 5.519 |
| SII | 0.122 | 8.198 |  | 0.124 | 8.087 |
| BMI (kg/m^2^) | 0.400 | 2.497 |  | 0.400 | 2.497 |
| WL (%) | 0.878 | 1.138 |  | 0.879 | 1.137 |
| MAC (cm) | 0.560 | 1.785 |  | 0.562 | 1.779 |
| TSF (mm) | 0.669 | 1.495 |  | 0.669 | 1.495 |
| CC (cm) | 0.638 | 1.567 |  | 0.639 | 1.564 |
| HGS (kg) | 0.536 | 1.866 |  | 0.536 | 1.865 |
| RRM.ASMI | 0.475 | 2.106 |  | 0.475 | 2.106 |
| RRM.FFMI | 0.856 | 1.168 |  | 0.857 | 1.167 |

CRP: C-reaction protein; NLR: neutrophils to lymphocytes ratio; PLR: platelets to lymphocytes ratio; SII: systematic inflammation index; BMI: body mass index; WL: weight loss within the first month; MAC: mid‐arm circumference; TSF: triceps skinfold thickness; CC: maximum calf circumference; HGS: hand grip strength; RMM: reduced muscle mass; ASMI: appendicular skeletal muscle mass index; FFMI: fat-free mass index.
